# Exploring the bias: how skin color influences oxygen saturation readings via Monte Carlo simulations

**DOI:** 10.1117/1.JBO.29.S3.S33308

**Published:** 2024-08-29

**Authors:** Suvvi K. Narayana Swamy, Chong Liu, Ricardo Correia, Barrie R. Hayes-Gill, Stephen P. Morgan

**Affiliations:** University of Nottingham, Optics and Photonics Research Group and Centre for Healthcare Technologies, Nottingham, United Kingdom

**Keywords:** pulse oximeter, oxygen saturation, transmission mode, occult hypoxemia, racial bias, melanin, skin color, Monte Carlo

## Abstract

**Significance:**

Our goal is to understand the root cause of reported oxygen saturation (${\mathrm{SpO}}_{2}$) overestimation in heavily pigmented skin types to devise solutions toward enabling equity in pulse oximeter designs.

**Aim:**

We aim to gain theoretical insights into the effect of skin tone on ${\mathrm{SpO}}_{2}\text{-}R$ curves using a three-dimensional, four-layer tissue model representing a finger.

**Approach:**

A finger tissue model, comprising the epidermis, dermis, two arteries, and a bone, was developed using a Monte Carlo-based approach in the MCmatlab software. Two skin tones—light and dark—were simulated by adjusting the absorption and scattering properties within the epidermal layer. Following this, ${\mathrm{SpO}}_{2}\text{-}R$ curves were generated in various tissue configurations, including transmission and reflection modes using red and infrared wavelengths. In addition, the influence of source–detector (SD) separation distances on both light and dark skin tissue models was studied.

**Results:**

In transmission mode, ${\mathrm{SpO}}_{2}\text{-}R$ curves did not deviate with changes in skin tones because both pulsatile and non-pulsatile terms experienced equal attenuation at red and infrared wavelengths. However, in reflection mode, measurable variations in ${\mathrm{SpO}}_{2}\text{-}R$ curves were evident. This was due to differential attenuation of the red components, which resulted in a lower perfusion index at the red wavelength in darker skin. As the SD separation increased, the effect of skin tone on ${\mathrm{SpO}}_{2}\text{-}R$ curves in reflection mode became less pronounced, with the largest SD separation exhibiting effects similar to those observed in transmission mode.

**Conclusions:**

Monte Carlo simulations have demonstrated that different light pathlengths within the tissue contribute to the overestimation of ${\mathrm{SpO}}_{2}$ in people with darker skin in reflection mode pulse oximetry. Increasing the SD separation may mitigate the effect of skin tone on ${\mathrm{SpO}}_{2}$ readings. These trends were not observed in transmission mode; however, further planned research using more complex models of the tissue is essential.

## 1 Introduction

Continuous and accurate monitoring of physiological changes is critical in medical settings, making peripheral oxygen saturation (${\mathrm{SpO}}_{2}$) a crucial vital sign in patient treatment and care.^1^ ${\mathrm{SpO}}_{2}$ serves as an indicator of the overall functioning of the body’s main organ systems, and its continuous observation is key for early problem detection and timely delivery of treatment and care. The gold standard for measuring arterial blood oxygen saturation (${\mathrm{SaO}}_{2}$) is arterial blood gas (ABG) sampling, a technique performed using a blood gas analyzer. This method is, however, intermittent, expensive, and time-consuming and involves invasive procedures. By contrast, pulse oximetry offers an alternative technology that is a continuous, inexpensive, pain-free, and non-invasive way of monitoring ${\mathrm{SpO}}_{2}$. This technique is realized using a pulse oximeter (PO), which operates by transmitting light into the tissue and measuring photoplethysmography (PPG) signals that represent light modulated by cardiac-synchronized variations in the arterial blood volume.^2^ Pulse oximeters (POs) commonly utilize red (660 nm) and infrared (IR, 940 nm) light wavelengths to measure the absorption of deoxyhemoglobin (Hbb) and oxyhemoglobin (${\mathrm{HbO}}_{2}$) in the blood, respectively. The basis of pulse oximetry is the “ratio of ratios” R of the pulsatile (AC)/non-pulsatile (DC) detected light components at red and IR wavelengths, obtained from PPG signals.^3^ The relationship between R and ${\mathrm{SaO}}_{2}$ is usually derived via calibration with ABG during controlled desaturation events.^4^ It is a simple, yet powerful technique in estimating continuous blood oxygen saturation readings in real time that has gained significant use in various clinical and remote patient monitoring settings.

For instance, during the COVID-19 pandemic, POs played a pivotal role in guiding clinicians and doctors in making timely clinical decisions, especially during patient triaging or when adjusting supplemental oxygen levels.^5^ Silent hypoxia, also known as “happy hypoxia,” was a common symptom experienced by people infected with this disease, wherein there was a sudden drop in ${\mathrm{SpO}}_{2}$ levels without any signs of deterioration in health.^6^ As a result, remote use of POs became widespread globally among COVID-19 patients or those at high risk to monitor their ${\mathrm{SpO}}_{2}$ levels regularly.^5^ Simultaneously, there was a rise in studies documenting POs inaccuracies, particularly in individuals with non-White skin tones.^7^^–^^10^ One large-scale retrospective study by Sjoding et al.^7^ highlighted that, when POs are utilized, Black patients were three times more likely to experience occult hypoxemia compared with those with White pigmentation. Occult hypoxemia is defined as a condition during which PO measures ${\mathrm{SpO}}_{2}>90\%$, despite ${\mathrm{SaO}}_{2}<88\%$ (i.e., overestimation). This discrepancy has since raised considerable alarm within the medical and scientific communities as overestimating ${\mathrm{SpO}}_{2}$ levels could hinder the timely and appropriate escalation of care delivered for those affected. Another extensive study conducted by Crooks et al.^11^ on patients at the time of transfer to the intensive care unit (ICU) confirmed that skin-tone-related measurement errors in pulse oximetry could lead to health and healthcare disparities across ethnic groups worldwide. Non-White subjects recorded similar ${\mathrm{SpO}}_{2}$ levels, but reduced ${\mathrm{SaO}}_{2}$ readings compared with White subjects and, as a result, were found to be more severely ill at the time of transfer to the ICU. This is consistent with the overestimation and delayed treatment hypothesis for heavily pigmented patients.

With the growing demand for improved PO reliability and accuracy across diverse skin tones, understanding how skin color influences ${\mathrm{SpO}}_{2}$ readings becomes crucial. Dark skin has a high melanin concentration that attenuates light greatly. As the basis of pulse oximetry is the ratio of ratios R, differences in melanin concentration should be compensated. However, the melanin absorbs and scatters light differently (red attenuation greater than IR),^12^ which may induce differences in R for different skin tones and therefore errors in ${\mathrm{SpO}}_{2}$ (as R is inversely proportional to ${\mathrm{SpO}}_{2}$).^13^ In our recent study, a laboratory benchtop simulator was constructed to generate PPG signals in a controlled manner, and the performance of commercially available home and hospital-based POs under the simulated effects of different levels of melanin in the skin were assessed.^14^^,^^15^ We hypothesized that high melanin concentration in the skin has two principal effects: (a) a reduced signal-to-noise ratio (SNR) and (b) preferential attenuation of red over IR light. These effects were simulated using neutral density and synthetic melanin filters. We discovered that none of the tested POs exhibited ${\mathrm{SpO}}_{2}$ overestimation under the effects of varying melanin attenuation. Although this study provided an opportunity to assess POs without the more complex ABG procedures that require testing on human volunteers, the use of a simplified simulator introduced limitations by not accounting for variations in optical properties that may occur in real-world situations (e.g., scattering). To further investigate this issue, numerical simulations such as Monte Carlo (MC) can be used to model both the scattering and absorption properties of melanin.

MC simulations have been extensively used to investigate light–tissue interactions across a wide range of PPG applications.^16^^,^^17^ The impact of skin tone on these interactions has been investigated in previous MC studies and revealed that increased melanin concentration reduces light intensity, resulting in a lower SNR.^18^^,^^19^ A study led by Arefin et al.^20^ showed that ${\mathrm{SpO}}_{2}$ overestimation in highly pigmented cohorts was minimized when the calibration equation was derived from a population cohort with a higher number of dark-pigmented models. Despite these efforts, a comprehensive physical understanding of the root cause of ${\mathrm{SpO}}_{2}$ overestimation due to skin color remains unknown. The primary aim of this paper is to address this by offering insights into the specific factors that may drive ${\mathrm{SpO}}_{2}$ overestimation in POs due to skin color variations, paying particular attention to the trajectories of the light paths at different wavelengths.

## 2 Methods

MCmatlab, an open-source software tool developed by Marti et al.,^21^ was utilized for carrying out MC simulations on a four-layer tissue model. These simulations aimed to investigate the impact of melanin’s optical properties on light–tissue interactions, ratio R and ${\mathrm{SpO}}_{2}$. This section outlines the geometry, parameters, and optical properties of the tissue model and the simulation methodology employed in this study.

### 2.1 Geometry of the Finger Model

**Fig. 1:**
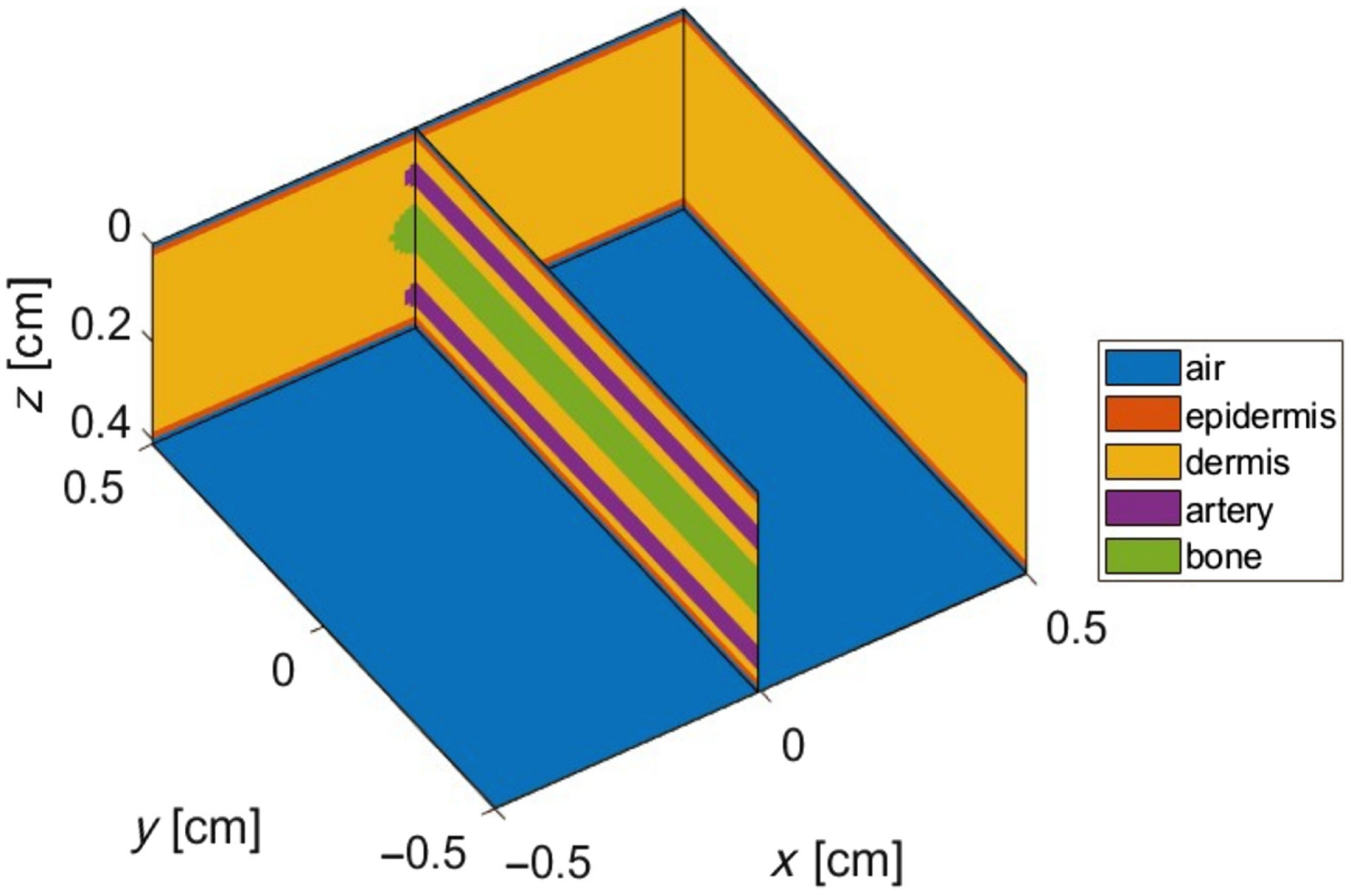
Output geometry of the finger tissue model produced by MCmatlab, containing the epidermis, dermis, two arteries, and a bone.^21^

A finger tissue model was constructed; it contained four layers: epidermis, dermis, two arteries, and a bone. The geometry of this four-layer tissue model is presented in Fig. 1. The overall structure of the geometry was modeled by a three-dimensional, semi-infinite cuboid model. The dimensions of the cuboid geometry were 1×1×0.41  cm (x,y,z), and it was divided into 101×101×201 bins in the (x,y,z) direction, with the finger thickness similar to that used in other work.^22^ The thickness of the various tissue layers included in the model is provided in Table 1.

**Table 1:** Thickness of tissue layers in the finger model.^22^^,^^23^

Layers	Thickness, cm
Epidermis	0.015
Dermis	0.36
Artery	0.025 (radius) at depths of 0.07 and 0.31
Bone	0.05 (radius) at a depth of 0.18

### 2.2 Tissue Parameters and Optical Properties

The parameters used to simulate the layers in Table 1, namely water volume ($V_{w}$), melanin volume ($V_{m}$), arterial blood volume ($V_{a}$), and venous blood volume ($V_{v}$), are all presented as fractions in Table 2. Two skin tones were simulated by adjusting $V_{m}$ in the epidermal layer to 0.05 (light skin) and 0.25 (dark skin), which correspond to Fitzpatrick type 2 (light) and type 5 (dark), respectively.^24^ The pulse was simulated by increasing $V_{a}$ during systole twice as high as in diastole. $V_{v}$ was maintained equal to the diastolic $V_{a}$ throughout simulations.^16^

**Table 2:** Parameters utilized for modeling tissue layers in the simulation. These values were adapted from the literature.^16^^,^^21^

Layers	$V_{w}$	$V_{m}$	$V_{a}$	$V_{v}$
Epidermis	0.2	0.05 (light)/0.25 (dark)	—	—
Dermis	0.3	—	0.02	0.02
Artery	0.6	—	0.3 (diastole)/0.6 (systole)	0.3

The two principal optical properties governing light attenuation through tissue are the absorption coefficient ($\mu_{a}$) and the scattering coefficient ($\mu_{s}$). These properties differ across tissue layers, depending on the type and concentration of chromophores present. To simulate variations in skin tone, both $\mu_{a}$ and $\mu_{s}$ values within the epidermal layer were altered. The $\mu_{a}$ of any tissue layer (denoted as the i’th layer) at a particular wavelength (λ) was determined as

$$\mu_{a_{i}}(\lambda )=V_{a_{i}}\mu_{a_{A_{i}}}(\lambda )+V_{v_{i}}\mu_{a_{V_{i}}}(\lambda )+V_{w_{i}}\mu_{a_{w_{i}}}(\lambda )+V_{m_{i}}\mu_{a_{m_{i}}}(\lambda )+[1-(V_{A_{i}}+V_{V_{i}}+V_{w_{i}}+V_{m_{i}})]\mu_{a_{\text{baseline}}}(\lambda )\;{\mathrm{cm}}^{-1},$$ (1)

where $\mu_{a_{\text{baseline}}}$ was defined by Eq. (2) and accounts for the absorption due to chromophores in the tissue other than the melanin, water, and blood.^16^

$$\mu_{a_{\text{baseline}}}(\lambda )=7.84\times 10^{8}\times \lambda^{-3.255}\;{\mathrm{cm}}^{-1}.$$ (2)

Parameters $\mu_{a_{A}}$, $\mu_{a_{V}}$, $\mu_{a_{m}}$, and $\mu_{a_{w}}$ are the absorption coefficients (${\mathrm{cm}}^{-1}$) of the arterial blood, venous blood, melanin, and water, respectively. Oxygen saturation is defined by the ratio of ${\mathrm{HbO}}_{2}$ concentration to the total Hb concentration present in the blood. Based on this definition, $\mu_{a_{A}}$ and $\mu_{a_{V}}$ are calculated using Eqs. (3) and (4), respectively.^16^ Absorption coefficients (${\mathrm{cm}}^{-1}$) of water, melanin, ${\mathrm{HbO}}_{2}$, and Hbb at 660 and 940 nm are presented in Table 3. ${\mathrm{SaO}}_{2}$ was varied from 60% to 100% in increments of 10%, and venous oxygen saturation (${\mathrm{SvO}}_{2}$) was maintained at 10% lower than the corresponding ${\mathrm{SaO}}_{2}$ values.^16^

$$\mu_{a_{A}}(\lambda )={\mathrm{SaO}}_{2}\mu_{a_{{\mathrm{HbO}}_{2}}}(\lambda )+(1-{\mathrm{SaO}}_{2})\mu_{a_{\mathrm{HHb}}}(\lambda )\;{\mathrm{cm}}^{-1},$$ (3)

$$\mu_{a_{V}}(\lambda )={\mathrm{SvO}}_{2}\mu_{a_{{\mathrm{HbO}}_{2}}}(\lambda )+(1-{\mathrm{SvO}}_{2})\mu_{a_{\mathrm{HHb}}}(\lambda )\;{\mathrm{cm}}^{-1},$$ (4)

where $\mu_{a}$ at 660 and 940 nm for the epidermis and bone layer^16^ are summarized in Table 4. Although $\mu_{a}$ values for the dermal and arterial layers are not listed in the table due to their dependency on oxygen saturation levels, they have been included in the simulation (with the values derived according to the steps outlined above). In addition, this table includes $\mu_{s}$ for all tissue layers at both wavelengths. Scattering is known to vary with an increase in melanin concentration,^27^ as a result, an estimate of the scattering coefficient was introduced in the epidermis to simulate different scattering properties associated with variations in skin tone.^23^

**Table 3:** Absorption coefficients of chromophores at specific pulse oximetry wavelengths.^25^

Absorption coefficient (${\mathrm{cm}}^{-1}$)	660 nm	940 nm
$\mu_{a_{m}}$	269.43	82.99
$\mu_{a_{w}}$	0.0036	0.2674
$\mu_{a_{{\mathrm{HbO}}_{2}}}$	1.71	6.50
$\mu_{a_{\mathrm{Hbb}}}$	17.27	3.71

**Table 4:** Optical properties of tissue layers.^16^^,^^23^^,^^26^

Layers	$\mu_{a}\;$ (${\mathrm{cm}}^{-1}$)		$\mu_{s}$ (${\mathrm{cm}}^{-1}$)	
	660 nm	940 nm	660 nm	940 nm
Epidermis	Light - 13.86	Light - 4.32	Light - 250	Light - 150
	Dark - 67.65	Dark - 20.89	Dark - 300	Dark -170
Dermis	—	—	279	194.7
Artery	—	—	75.76	53.19
Bone	0.351	0.457	344.5	247

For calculating the $\mu_{s}$ for the dermal and arterial layers, at first, reduced scattering coefficient ($\mu_{s}^{\prime }$) values that accounts for the anisotropy of scattering (g) were calculated as^26^

$$\mu_{s}^{\prime }(\lambda )=a^{\prime }{\left(f_{\text{Ray}}(\frac{\lambda }{500\;\mathrm{nm}})\right)}^{-4}+(1-f_{\text{Ray}}){\left(\frac{\lambda }{500\;\mathrm{nm}}\right)}^{-b_{\text{Mie}}}\;{\mathrm{cm}}^{-1},$$ (5)

where $f_{\text{ray}}$ and $b_{\text{Mie}}$ correspond to Rayleigh and Mie scattering coefficients, respectively, and $a^{\prime }$ denotes a scaling factor. These parameters were obtained from existing literature for the dermis and arterial layer and are summarized in Table 5. Finally, $\mu_{s}$ was estimated by scaling $\mu_{s}^{\prime }$ by a factor of (1-g).

**Table 5:** Parameters used to calculate reduced scattering coefficient for the dermal and arterial layers.^26^

Layers	$a^{\prime }$ (${\mathrm{cm}}^{-1}$)	$f_{\text{ray}}$	$b_{\text{Mie}}$
Dermis	43.6	0.41	0.562
Artery	10	—	1

For simplification, all tissue layers in the model were assigned a uniform refractive index (n=1.43) and anisotropy factor (g=0.9), so reflections at the air-tissue and tissue-tissue boundaries are not taken into account. The primary aim was to study ${\mathrm{SpO}}_{2}$ overestimation due to skin tone by isolating the effect of epidermal absorption and scattering properties on light–tissue interactions from other complex interactions, so a simple model was desirable.

### 2.3 Simulation Procedure

**Fig. 2:**
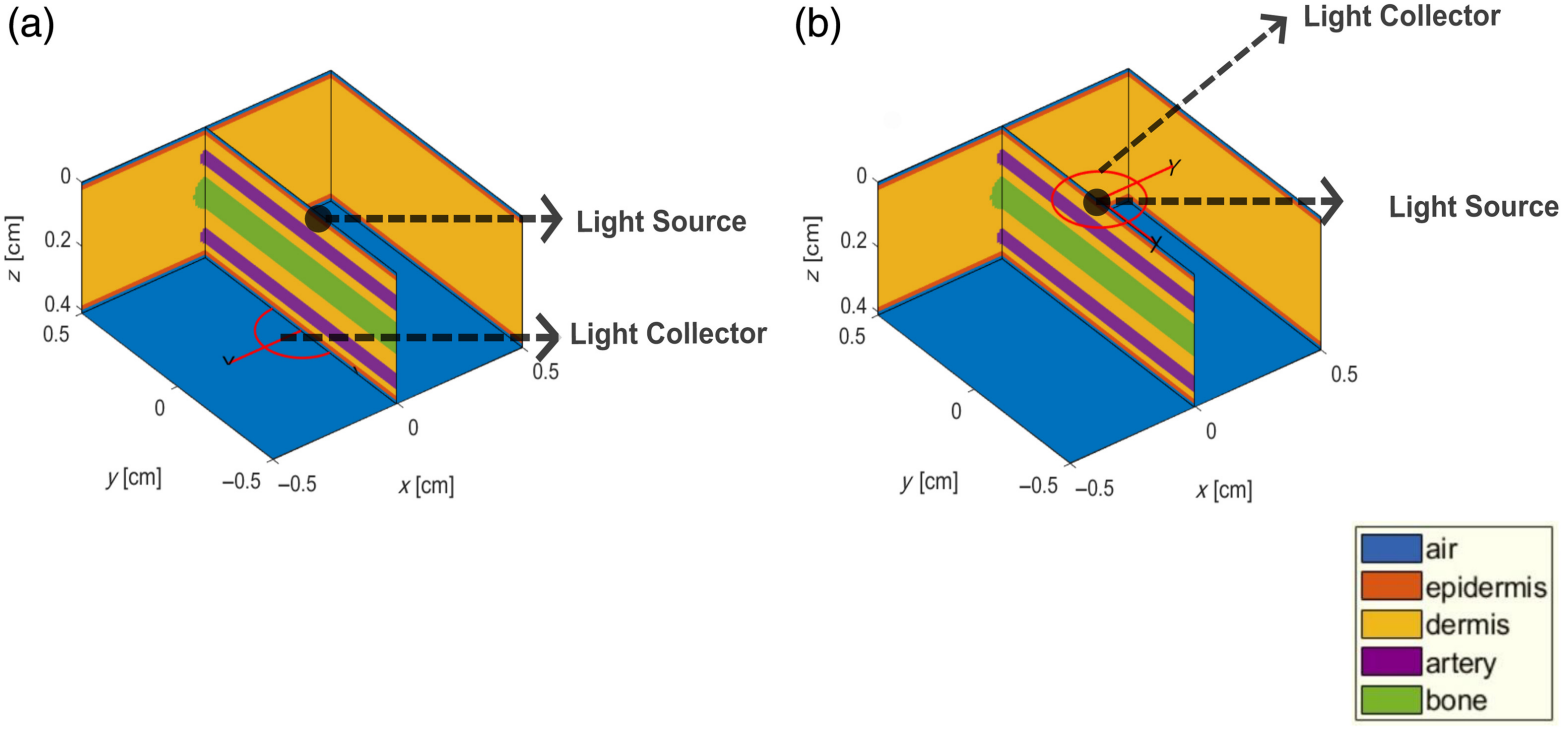
Positioning and placement of the light source and collector illustrated in (a) transmission mode and (b) reflection mode geometry. The source is represented by a black dot, and the detector is represented by a red ring.

Transmission and reflection modes were simulated with the source and detector were either positioned on opposite sides of the tissue (transmission) or adjacent to each other on the same side of the tissue (reflection). The position and placement of the light source and collector in transmission and reflection modes are depicted in Figs. 2(a) and 2(b), respectively. The simulations were run on a remote desktop with Windows 10 Enterprise, an Intel Xeon CPU E7 with 40 cores and 1 TB of main memory. A Gaussian beam with a radial width of 0.2 cm simulated the illumination at the tissue surface. In commercial POs, the detector is usually a photodiode. The most effective way of implementing this in MCmatlab was by simulating this as a fiber optic collector with a diameter of 0.3 cm and a numerical aperture of 1. For each simulation run, a total of $5\times 10^{7}\text{ photons}$ were simulated, and this process was repeated three times to ensure repeatability and consistency in the measured results. After the execution of the MC simulation, various outputs could be extracted during systolic and diastolic states at 660 and 940 nm such as the normalized DC light intensities from the light collector (${\mathrm{DC}}_{\text{diastole}}$ and ${\mathrm{DC}}_{\text{systole}}$), normalized fluence rate distribution of the detected photons within the tissue, optical path length (OPL), and penetration depth (PD, only in reflection mode). The AC pulsatile intensity was determined from the difference in DC intensities between diastolic and systolic states. The perfusion index, denoted by ($PI_{\lambda }$), was defined as the ratio of the AC pulsatile intensity to the DC diastolic intensity:

$${\mathrm{PI}}_{\lambda }=\frac{{\mathrm{AC}}_{\lambda }}{{\mathrm{DC}}_{{\text{diastole}}_{\lambda }}}.$$ (6)

Finally, R was computed by taking the ratio of PIs at red and IR wavelengths, given as

$$R=\frac{{\mathrm{PI}}_{\text{red}}}{{\mathrm{PI}}_{\mathrm{IR}}}.$$ (7)

To aid the comparison, we also defined attenuation factors that describe the relative responses of light and dark skin, where a is the relative AC attenuation of dark skin to light skin at the red wavelength, b is the relative DC attenuation of dark skin to light skin at the red wavelength, c is the relative AC attenuation of dark skin to light skin at the IR wavelength, and d is the relative DC attenuation of dark skin to light skin at the IR wavelength. This leads to a ratio of ratios R for dark skin, given as

$$R_{\text{dark}}=\frac{\frac{a{\mathrm{AC}}_{\text{red}}}{b{\mathrm{DC}}_{\text{red}}}}{\frac{c{\mathrm{AC}}_{\mathrm{IR}}}{d{\mathrm{DC}}_{\mathrm{IR}}}}.$$ (8)

The median PD of the detected photons was defined as the median of the depths (i.e., the maximum distance traveled along the z-axis). Similarly, the median OPL of the detected photons was defined as the median of the total OPL (i.e., the sum of all N step sizes corresponding to the number of scattering and absorption events). It should be noted that the PD and OPL data were tested for normality using the Kolmogorov-Smirnov (KS) test in MATLAB. The findings revealed that the data was not normally distributed for both cases; hence the median (IQR) was chosen as the appropriate measure to describe the OPL and PD in this article.

## 3 Results

This section presents the results investigating the influence of skin color on ${\mathrm{SpO}}_{2}$ versus R curves, using both transmission and reflection mode configurations. In addition, it examines the impact of source–detector (SD) separation on light and dark skin tissue models.

To simulate the effect of skin color in Secs. 3.1–3.3, the absorption and scattering coefficients for the epidermal layer, as presented in Table 4, were varied. In addition, Table 4 contains the optical properties used for modeling different tissue layers. The oxygen saturation was varied from 60% to 100% in increments of 10% for each skin type in both the dermal and arterial layers. However, the pulse was only simulated in the arterial layer by doubling the blood volume in the systolic state compared with the diastolic state. The absorption coefficient for both the dermal and arterial layers was determined using the values in Table 3 in Eq. (1), and the scattering coefficient was calculated from Eq. (5) using the values in Table 5. A total of $5\times 10^{7}\text{ photons}$ were simulated during the diastolic and systolic phases at red and IR wavelengths, and this process was repeated three times.

### 3.1 Transmission Mode

In transmission mode, the light source was positioned at the origin, and the collector was placed at a distance of 0.41 cm, on the opposite side of the tissue surface [see Fig. 2(a)].

**Fig. 3:**
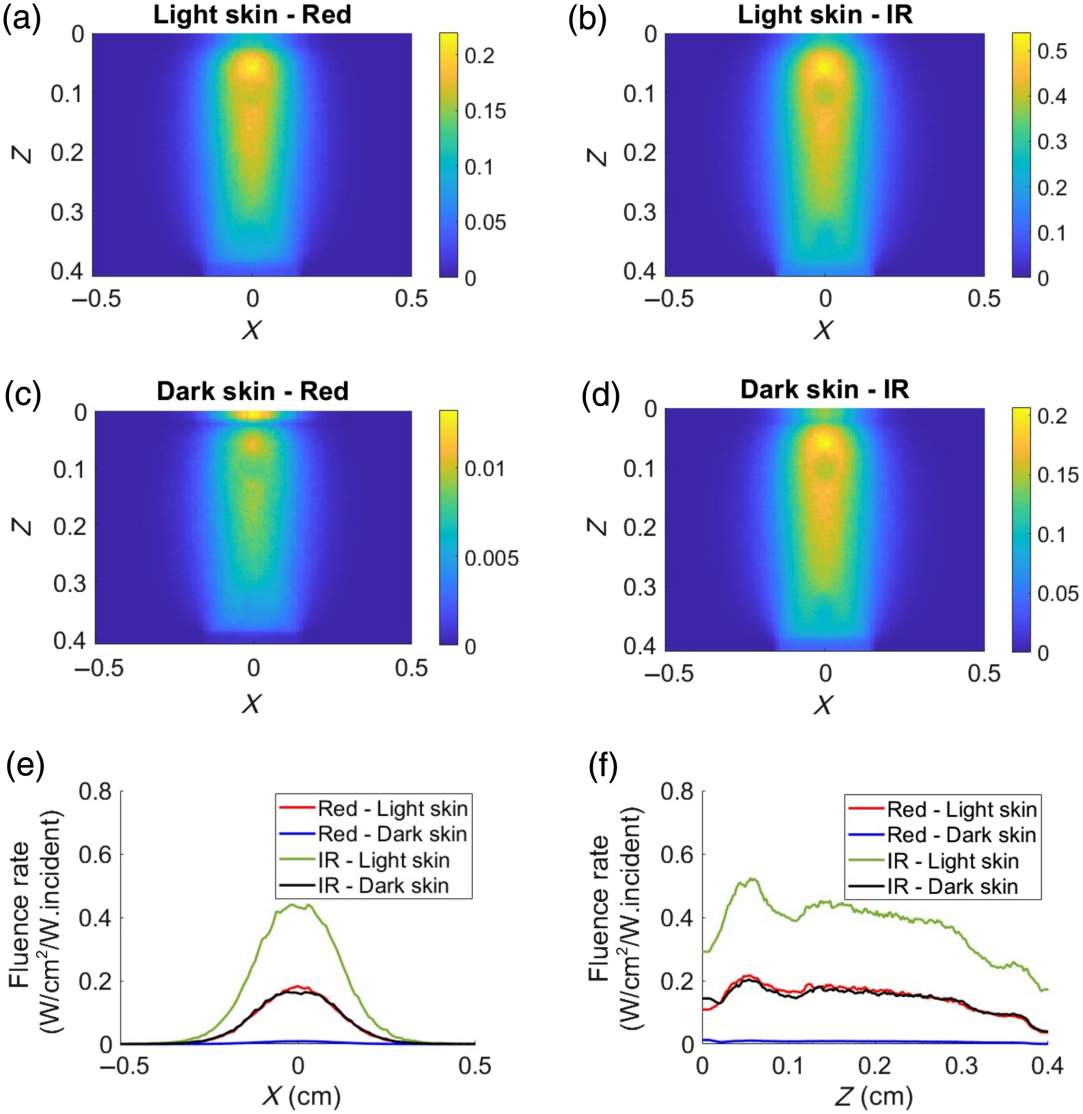
Normalized fluence rate distribution of the detected photons at ${\mathrm{SpO}}_{2}=90\%$ in transmission mode, shown at diastolic red and IR for light skin [(a), (b)] and for dark skin [(c), (d)], respectively. The color bar represents the magnitude of the distribution of detected photons through the finger tissue. Plots (e) and (f) show horizontal and vertical line profiles of the normalized fluence distribution, at Z=0.12  cm and X=0  cm, respectively.

A two-dimensional normalized fluence rate distribution that illustrates the magnitude of detected photon distribution within the tissue at an oxygen saturation value of 90% for both light and dark skin at different wavelengths is shown in Fig. 3. IR light, being the longer wavelength, exhibits a higher fluence rate distribution within the tissue (due to relatively lower light absorption) compared with the shorter red light in both skin types [seen from Figs. 3(b) and 3(d)]. Furthermore, the absorption of red light is more pronounced in dark skin, affecting its light distribution (lower fluence rate) within the tissue [observed in Figs. 3(a) and 3(c)]. The horizontal and vertical line profiles, drawn at Z=0.12  cm and X=0  cm, respectively, for both skin types corresponding to the two wavelengths of interest, are presented in Figs. 3(e) and 3(f). The maximum fluence rate ($W/{\mathrm{cm}}^{2}/W.\text{incident}$) within the tissue is found in the following order: IR, light skin (0.44) > red, light skin (0.18) > IR, dark skin (0.16) > red, dark skin ($9\times 10^{-3}$). From Fig. 3(f), the fluence rate decreased with increasing tissue depth. The highest fluence rate ($W/cm^{2}/W.\text{incident}$) at the collector facing the tissue boundary (Z=0.4  cm) is found in the following order: IR, light skin $(1.7\times 10^{-1})>\mathrm{IR}$, dark skin $(3.9\times 10^{-2})>\text{red}$, light skin $(3.6\times 10^{-2})>\text{red}$, dark skin ($6.86\times 10^{-4}$). This reveals that the intensity of light reaching the detector in transmission mode is highest for the IR wavelength in both skin types compared with the intensity at the red wavelength.

**Fig. 4:**
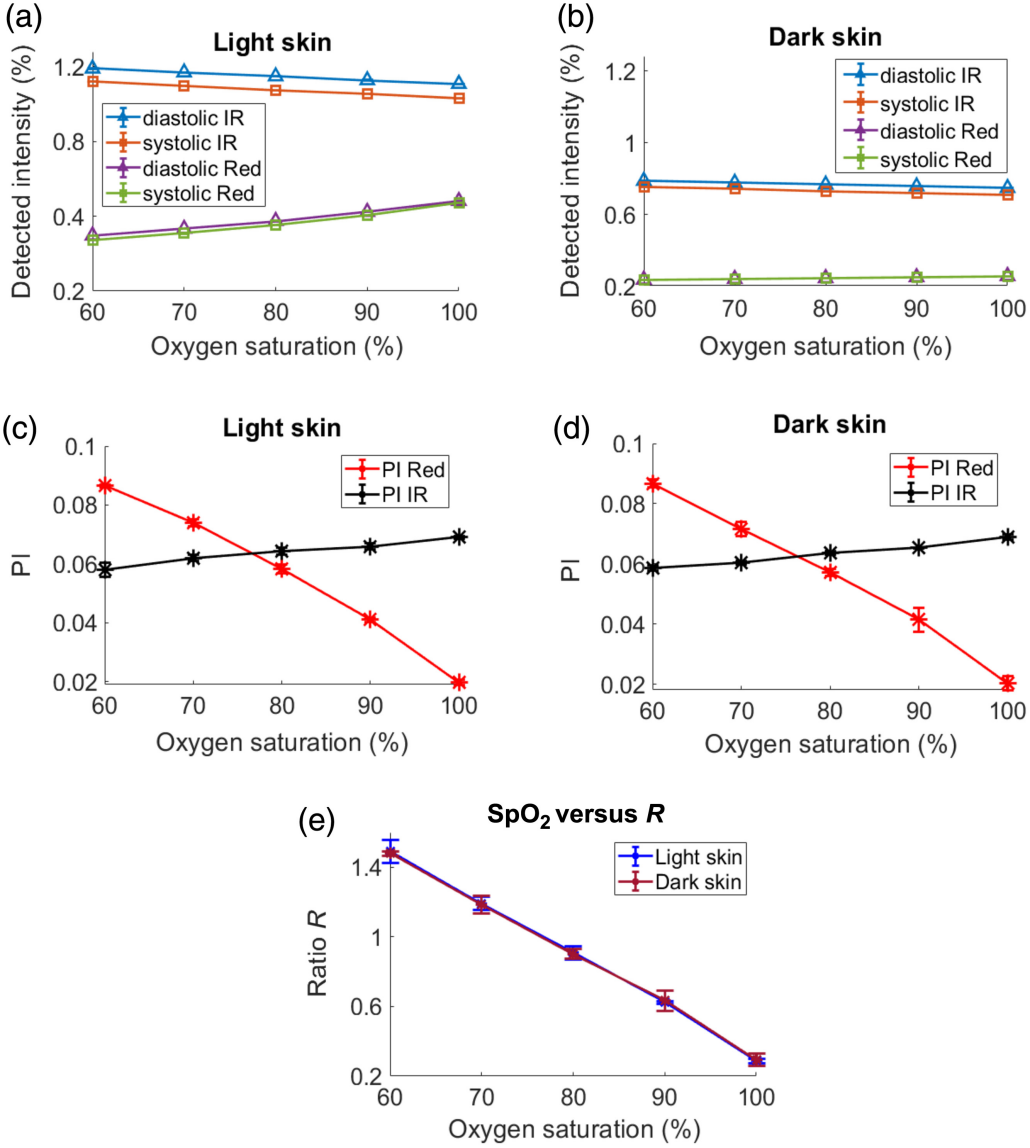
Detected light intensities in transmission mode tissue geometry for light and dark skin plotted against oxygen saturation and shown in panels (a) and (b), respectively. The PI at red and IR for light and dark skin as a function of oxygen saturation is illustrated in panels (c) and (d). The R ratio versus ${\mathrm{SpO}}_{2}$ curves for light and dark skin are presented in panel (e).

Figure 4 presents the detected light intensities, PIs, and ${\mathrm{SpO}}_{2}\text{-}R$ curves for light and dark skin tissue models in transmission mode. The overall transmitted light intensity was found to be higher for light skin compared with dark skin at both wavelengths [Figs. 4(a) and 4(b)]. However, in dark skin, the relative attenuation at the red wavelength was greater compared with the IR wavelength. For instance, at ${\mathrm{SpO}}_{2}=90\%$, the relative attenuation in dark skin at diastolic red was 89%, whereas at the diastolic IR, it was only 50% compared with light skin. Throughout this paper, trends at ${\mathrm{SpO}}_{2}=90\%$ were selected as an illustrative example as it represents a clinically significant threshold for diagnosing hypoxemia^28^ and may indicate an urgent need for critical medical attention. Further, from Figs. 4(a) and 4(b), it is observed that the red-light intensity gradually increased with an increase in ${\mathrm{SpO}}_{2}$, whereas the IR light intensity showed a gradual decrease with an increase in saturation. This is expected as the molar extinction coefficient of Hbb is higher than ${\mathrm{HbO}}_{2}$ in the red, and in the IR, the opposite is true. For both light and dark skin, the diastolic light intensities were consistently higher than their systolic counterparts due to lower volumes of absorbing blood. The calculated PI values at red and IR for light and dark skin are displayed in Figs. 4(c) and 4(d), respectively. To quantify the overall difference or similarity in PI curves between skin types, the pairwise Euclidean distance was calculated; it quantifies the absolute difference in the magnitude for each wavelength. This method is consistently applied throughout the analysis. The difference in PIs at each wavelength between light and dark skin types was similar, as indicated by their Euclidean distances: $1\times 10^{-2}\;\mathrm{cm}$ for red PI and $1\times 10^{-2}\;\mathrm{cm}$ for IR PI.

For example, at an ${\mathrm{SpO}}_{2}=90\%$, the mean (standard deviation, std) of PI in red is $4.1\times 10^{-2}$ ($7\times 10^{-4}$) in light skin and $4.1\times 10^{-2}$ ($4\times 10^{-3}$) in dark skin. Similarly, at the same saturation, the mean (std) of PI at IR is $6.5\times 10^{-2}$ ($1\times 10^{-3}$) in light skin and $6.5\times 10^{-2}$ ($2.2\times 10^{-4}$) in dark skin. The ratio R for both skin types as a function of ${\mathrm{SpO}}_{2}$ is illustrated in Fig. 4(e). To evaluate the statistical significance between ${\mathrm{SpO}}_{2}\text{-}R$ curves in this paper, the Wilcoxon signed rank test, a non-parametric statistical test for paired data, was utilized with a significance level set at p<0.05. No measurable difference in ${\mathrm{SpO}}_{2}$ was observed between the two skin types under this configuration (p-value=0.84).

**Fig. 5:**
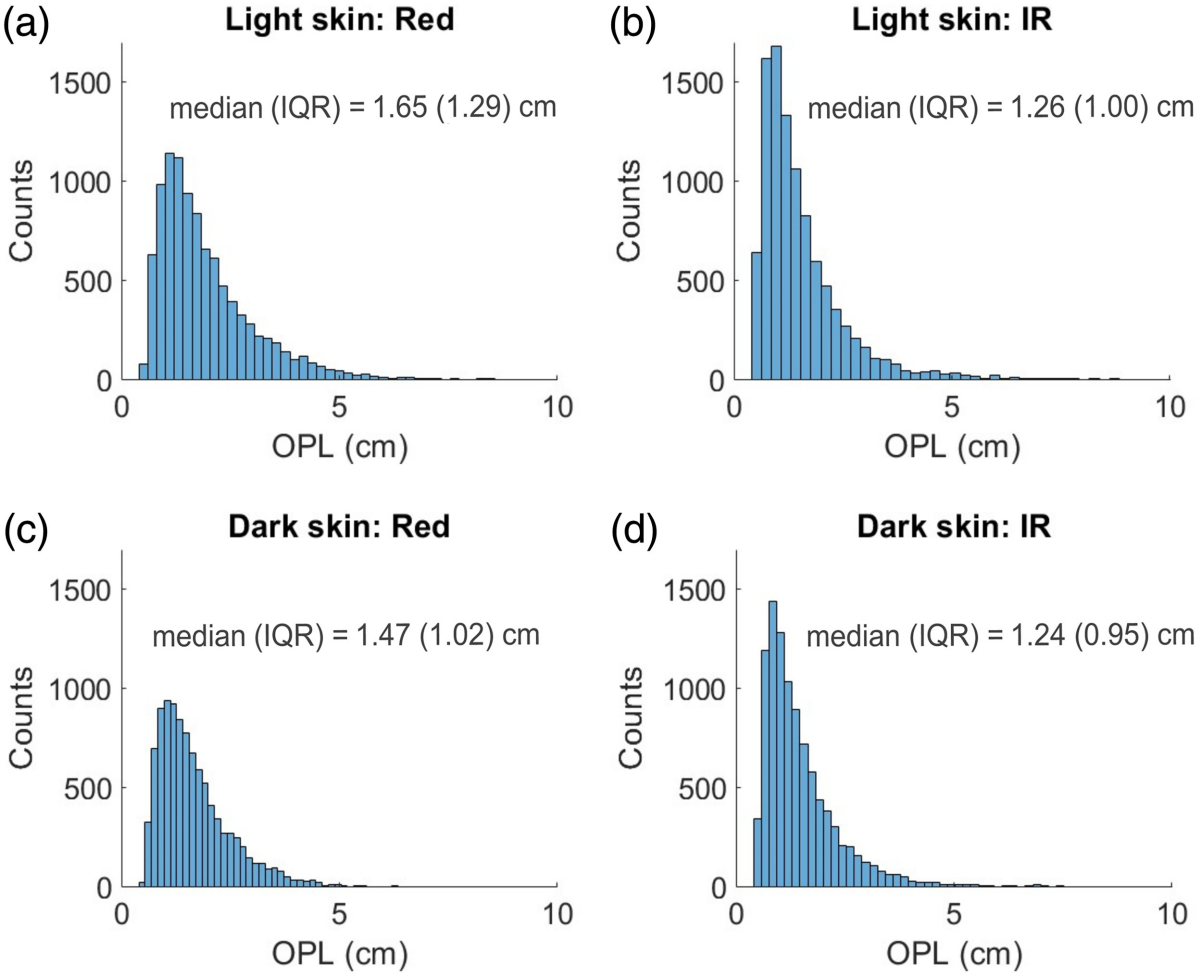
Distributions of OPL at diastolic red and IR wavelengths, presented for light skin [(a), (b)] and dark skin [(c), (d)]. These distributions are based on a sample of 10,000 detected photons in each case.

Figure 5 displays a histogram of OPL distributions based on a sample of 10,000 detected photons at both red and IR diastolic wavelengths for light and dark skin with an ${\mathrm{SpO}}_{2}$ of 90%. The OPL quantifies the total distance traveled by each detected photon within the tissue. The plot reveals a slight reduction in OPL for dark skin at the red wavelength, with the median (IQR) decreasing from 1.65 (1.29) to 1.47 (1.02) cm. At the IR, the median (IQR) OPL for dark skin is 1.26 (1.0) cm, which is comparable to the median (IQR) OPL for light skin at 1.24 (0.95) cm.

### 3.2 Reflection Mode

**Fig. 6:**
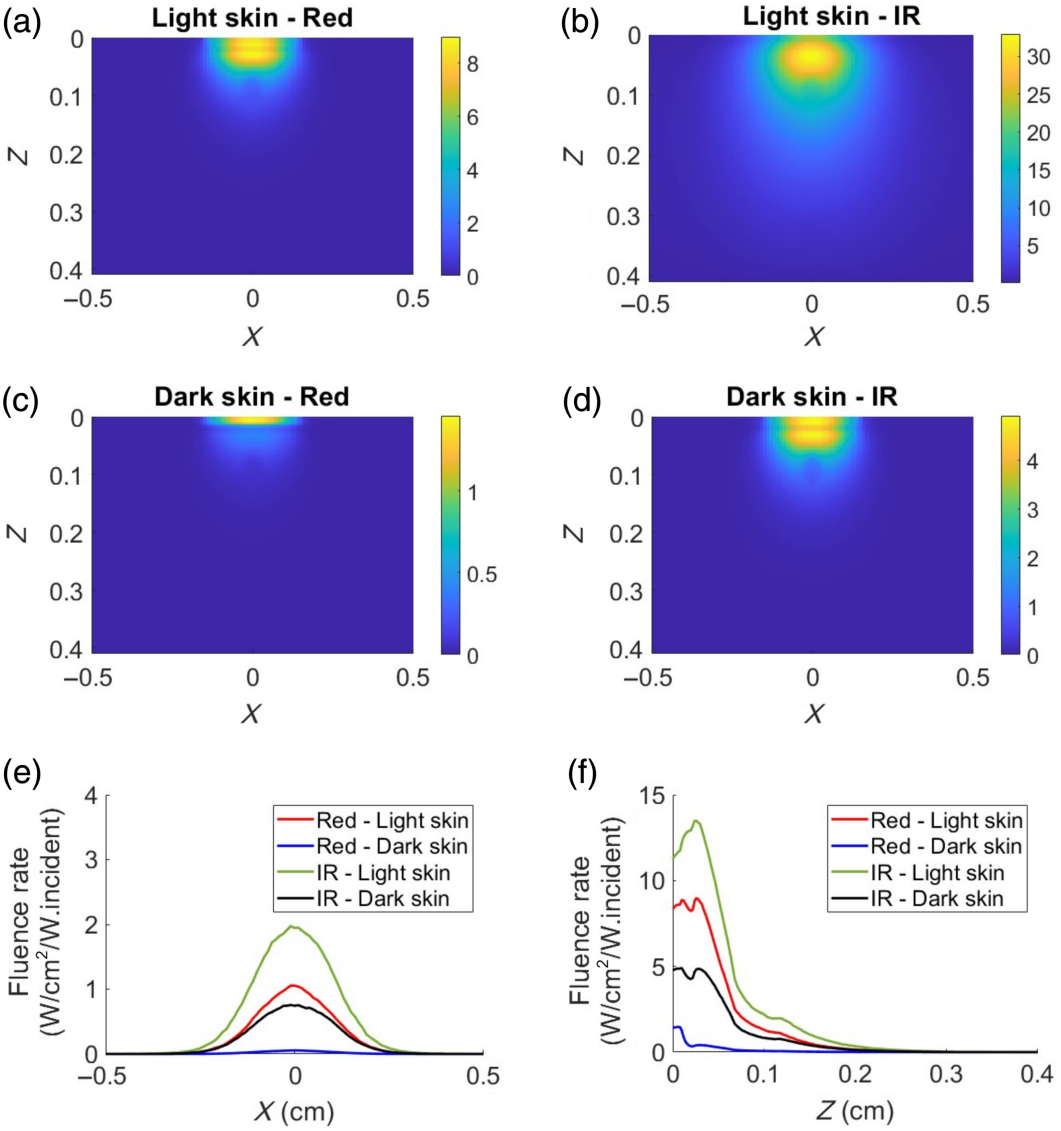
Normalized fluence rate distribution of the detected photons at ${\mathrm{SpO}}_{2}=90\%$ in reflection mode, shown at diastolic red and IR for light skin (a), (b) and for dark skin (c), (d), respectively. The color bar represents the magnitude of the distribution of detected photons through the finger tissue. Plots (e) and (f) show horizontal and vertical line profiles of the normalized fluence distribution, at Z=0.12  cm and X=0  cm, respectively.

In reflection mode, the light source was located at the origin, and the detector was slightly offset from the origin at (0, 0, $-1\times 10^{-4}$) to simulate a case of central illumination and detection [see Fig. 2(b)]. Figure 6 shows normalized fluence rate plots of the detected photons in reflection mode at an oxygen saturation of 90%, revealing similar observations to those seen in transmission mode (Fig. 3). Here, the increased absorption of red light in darker skin leads to reduced light penetration within the tissue. As seen in the transmission mode case, horizontal and vertical line profiles drawn at Z=0.12  cm and X=0  cm, respectively, for both skin types corresponding at the two wavelengths are presented in Figs. 6(e) and 6(f). The magnitude of the fluence rate ($W/cm^{2}/W.\text{incident}$) is higher for the IR wavelength compared with the red wavelength. For instance, at a depth of 0.1 cm, the maximum fluence rate is as follows: IR, light skin (2.19)>red, light skin (1.3)>IR, dark skin (0.83)>red, dark skin (0.06). This implies that the tissue penetration depth is greatest in IR for light skin, followed by red for light skin, IR for dark skin, and red for dark skin.

**Fig. 7:**
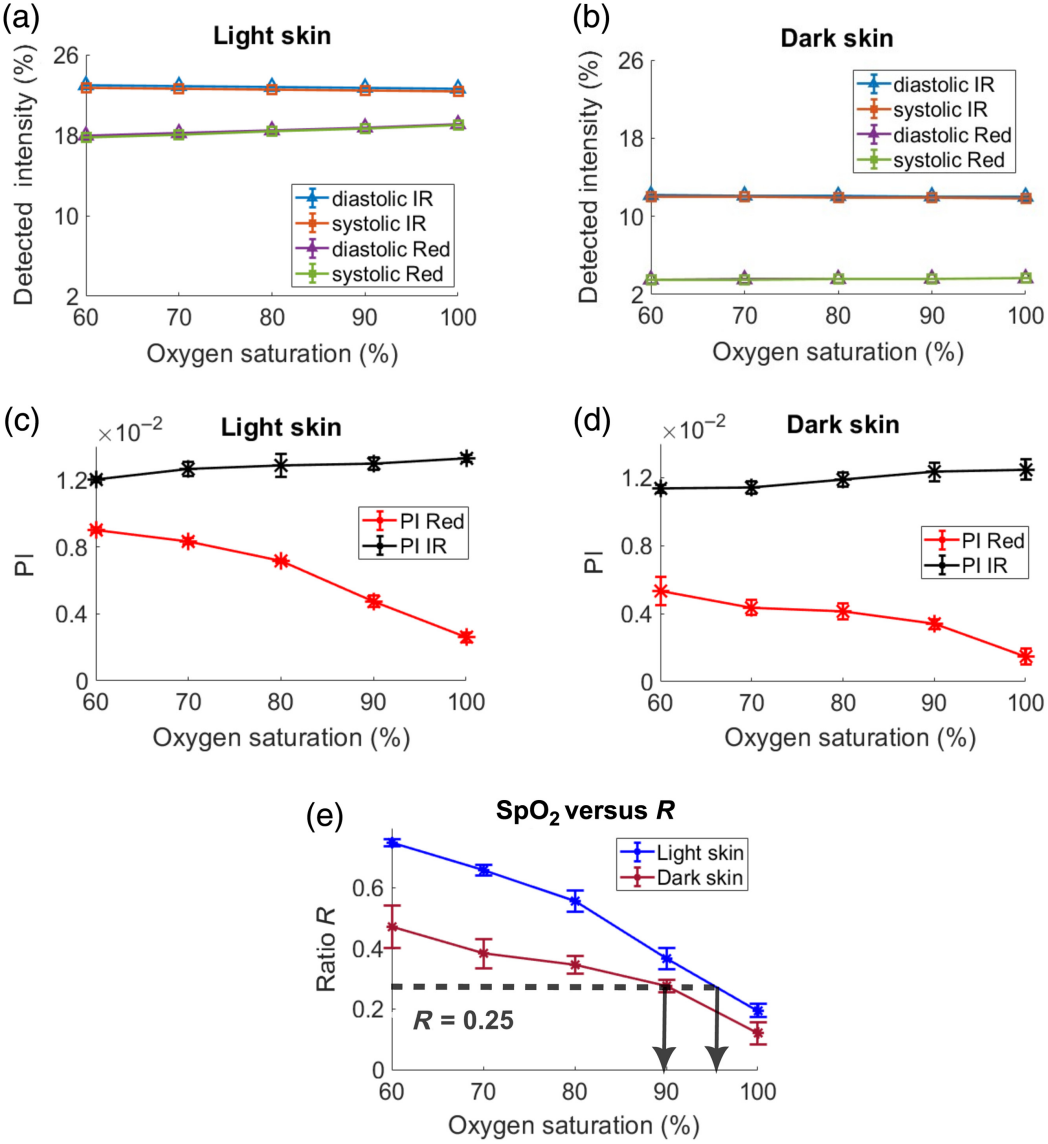
Detected light intensities in reflection mode (central illumination and detection) tissue geometry for light and dark skin plotted against oxygen saturation and shown in panels (a) and (b), respectively. The PI at red and IR for light and dark skin as a function of oxygen saturation is illustrated in panels (c) and (d). The R ratio versus ${\mathrm{SpO}}_{2}$ curves for light and dark skin are presented in panel (e).

The detected light intensities, PIs, and ${\mathrm{SpO}}_{2}\text{-}R$ curves for light and dark skin tissue models in reflection mode are presented in Fig. 7. Although the detected light intensities in reflection mode were significantly higher compared with those in transmission mode (36 times higher for IR and 18 times higher for red at an ${\mathrm{SpO}}_{2}$ of 90%), the observed trends with varying oxygen saturation for both light and dark skin, as illustrated in Figs. 7(a) and 7(b), show similar characteristics to the transmission mode plots [Figs. 4(a) and 4(b)]. Interestingly, as observed from Figs. 7(c) and 7(d), variations in skin tone did not have a large effect on the PI at IR wavelengths but caused a significant reduction in the red PI for darker skin tones across varying ${\mathrm{SpO}}_{2}$ levels. This is confirmed by the very small Euclidean distance between the PIs of light and dark skin at IR ($4\times 10^{-3}\;\mathrm{cm}$) compared with the larger Euclidean distance at red ($1.1\times 10^{-2}\;\mathrm{cm}$). As an example, at ${\mathrm{SpO}}_{2}=90\%$, the mean (std) of PI at red is $4.7\times 10^{-3}$ ($3.3\times 10^{-4}$) in light skin, which decreased to $3.4\times 10^{-3}$ ($2.9\times 10^{-4}$) in dark skin. At the same saturation, the mean (std) of the PI at IR is $1.3\times 10^{-2}$ ($3.6\times 10^{-4}$) in light skin and $1.2\times 10^{-2}$ ($5.5\times 10^{-4}$) in dark skin.

Contrary to the ${\mathrm{SpO}}_{2}\text{-}R$ curves seen in transmission mode, the reflection mode shows significant variations between light and dark skin, as seen in Fig. 7(e) ($p\text{-value}=6\times 10^{-5}$). For instance, at an R value of 0.25 (dashed horizontal line), the actual measured ${\mathrm{SpO}}_{2}$ for dark skin is 90%, which is lower compared with a value exceeding 90% shown for light skin. This indicates ${\mathrm{SpO}}_{2}$ overestimation in dark skin if a calibration curve originally derived from light skin is used.

### 3.3 Varying Source–Detector (SD) Separation—Reflection Mode

**Fig. 8:**
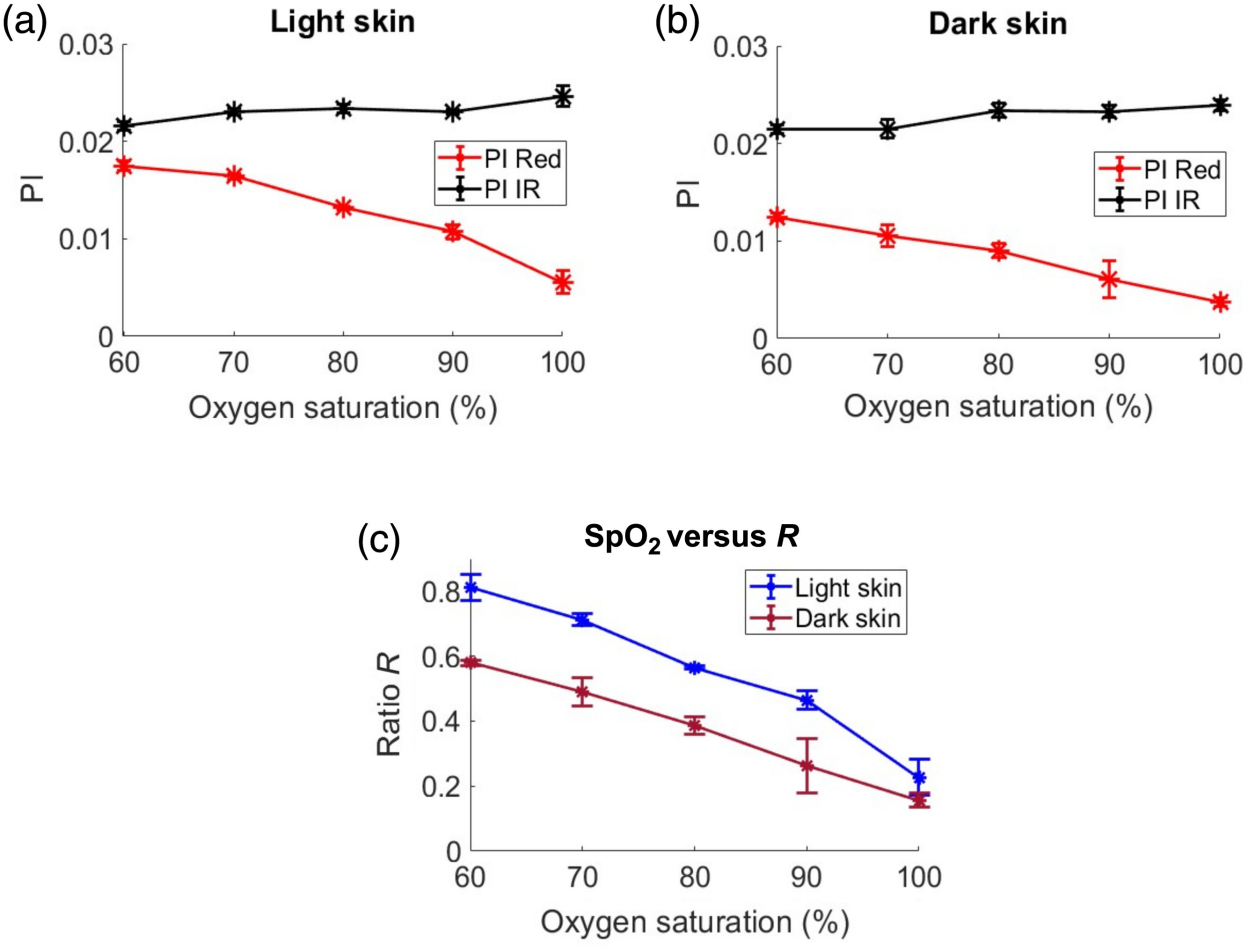
PI plots for light (a) and dark skin (b) as a function of ${\mathrm{SpO}}_{2}$ (%), along with its corresponding ${\mathrm{SpO}}_{2}\text{-}R$ curve (c), for a small SD distance.

**Fig. 9:**
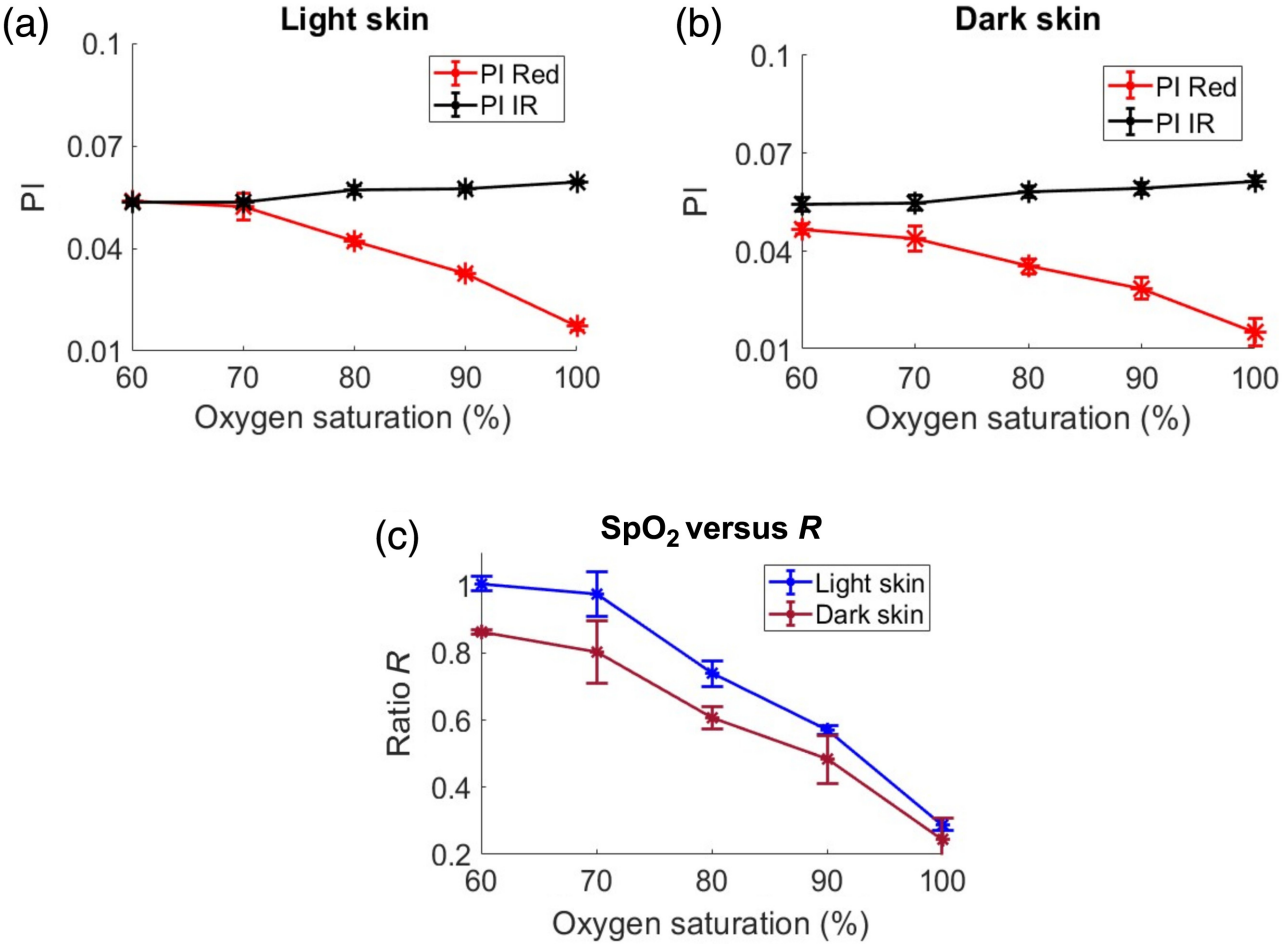
PI plots for light (a) and dark skin (b) as a function of ${\mathrm{SpO}}_{2}$ (%), along with its corresponding ${\mathrm{SpO}}_{2}\text{-}R$ curve (c), for a medium SD distance.

**Fig. 10:**
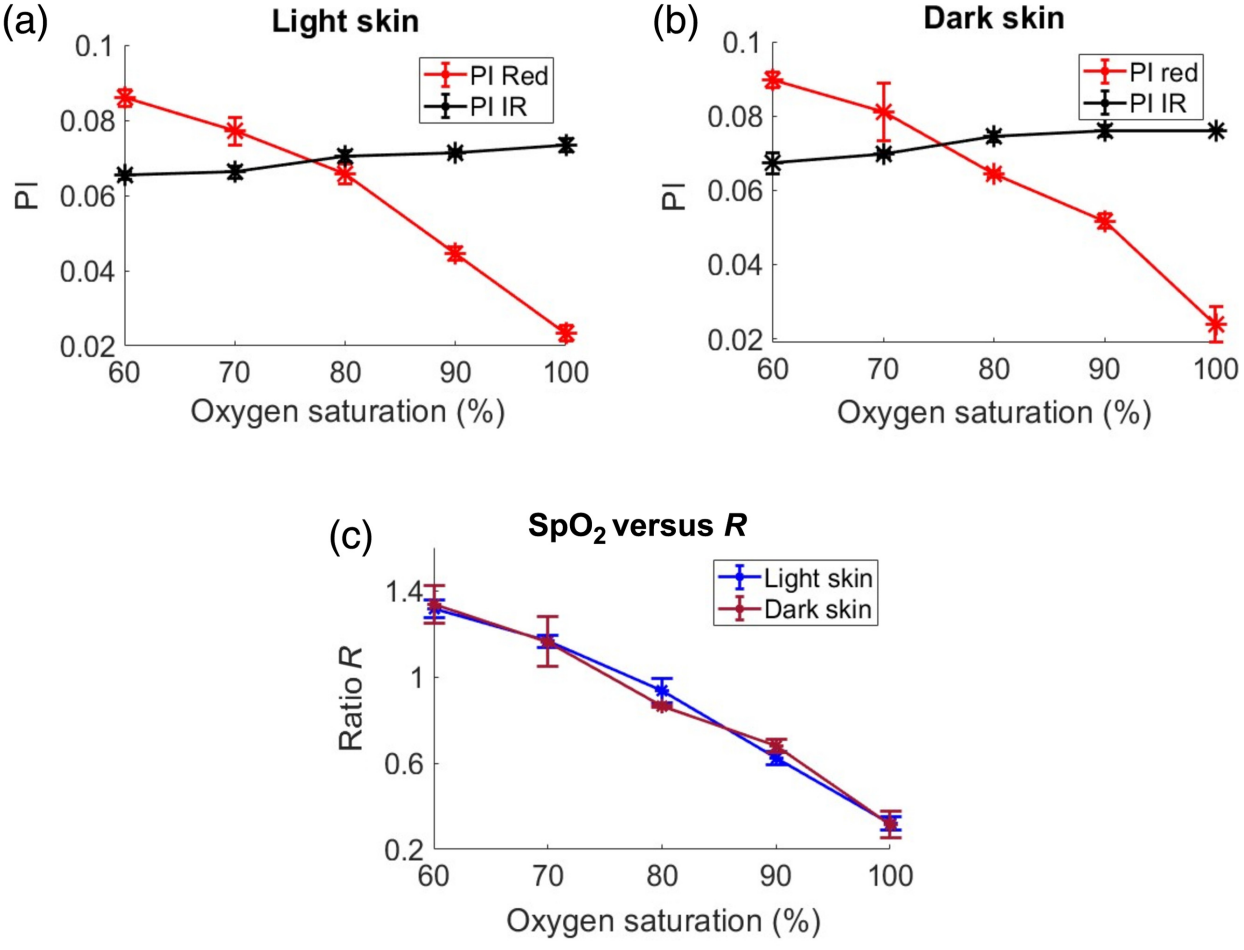
PI plots for light (a) and dark skin (b) as a function of ${\mathrm{SpO}}_{2}$ (%), along with its corresponding ${\mathrm{SpO}}_{2}\text{-}R$ curve (c), for a large SD distance.

To further explore the effects of varying SD separation on light and dark skin tissue models, simulations were conducted at three different separations: small (0.1 cm), medium (0.25 cm), and large (0.45 cm). As the SD separation increased, the PI values at IR and red also rose in both light and dark skin types due to greater DC light attenuation with larger SD separations, as depicted in Figs. 8(a), 8(b), 9(a), 9(b), 10(a), and 10(b). The IR PI values showed minimal differences between light and dark skin types for all SD separations considered (Euclidean distance: small $\mathrm{SD}=5\times 10^{-3}\;\mathrm{cm}$, medium $\mathrm{SD}=8\times 10^{-3}\;\mathrm{cm}$, and large $\mathrm{SD}=1.6\times 10^{-2}\;\mathrm{cm}$). However, slightly larger variations in red PI values were seen between light to dark skin due to the change in SD separation (Euclidean distance: small $\mathrm{SD}=1.8\times 10^{-2}\;\mathrm{cm}$, medium $\mathrm{SD}=2.6\times 10^{-2}\;\mathrm{cm}$, and large $\mathrm{SD}=2.4\times 10^{-2}\;\mathrm{cm}$). At a smaller SD distance, dark skin exhibited a relatively lower PI with a mean (std) of $6\times 10^{-3}$ ($2\times 10^{-3}$) at red compared with light skin PI of $1\times 10^{-2}$ ($7\times 10^{-4}$) at 90% saturation, as shown in Figs. 8(a) and 8(b). At the largest SD separation, the red and IR PIs in both light and dark skin became comparable [as shown in Figs. 10(a) and 10(b)] with Euclidean distances of $1.2\times 10^{-2}$ and $1\times 10^{-2}\;\mathrm{cm}$, respectively, at 90% saturation. Further, the variations in ${\mathrm{SpO}}_{2}\text{-}R$ curves between light and dark skin became less pronounced as the SD separation increased, as illustrated in Figs. 8(c), 9(c), and 10(c). This trend is statistically confirmed by the increasing p-values found with greater SD separations (p-value: small $\mathrm{SD}=1.2\times 10^{-4}$, medium $\mathrm{SD}=1.8\times 10^{-4}$, and large SD=0.97).

The decrease in intensity in dark skin relative to light skin, expressed as a fraction, was calculated at a particular ${\mathrm{SpO}}_{2}$ value of 90% to compare relative attenuation factors under different simulated configurations. This is summarized in Table 6. Mathematically, attenuation in dark skin with respect to light skin is expressed as shown in Eq. (8).

**Table 6:** Summary of AC and DC relative attenuation factors in dark skin with respect to light skin at red and IR wavelengths for ${\mathrm{SpO}}_{2}=90\%$ across different configurations.

Configuration type	a	b	c	d
Transmission	0.89 ($1.2\times 10^{-2}$)	0.89 ($2\times 10^{-4}$)	0.51 ($8.3\times 10^{-3}$)	0.51 ($5\times 10^{-4}$)
Reflection (central illumination and detection)	0.86 ($4.5\times 10^{-3}$)	0.81 ($4\times 10^{-5}$)	0.49 ($3.3\times 10^{-2}$)	0.47 ($9\times 10^{-5}$)
Reflection (small SD separation)	0.91 ($2.6\times 10^{-2}$)	0.83 ($1\times 10^{-4}$)	0.49 ($1.6\times 10^{-2}$)	0.50 ($2\times 10^{-4}$)
Reflection (medium SD separation)	0.90 ($1.5\times 10^{-2}$)	0.88 ($5\times 10^{-4}$)	0.52 ($1.9\times 10^{-2}$)	0.53 ($1\times 10^{-3}$)
Reflection (large SD separation)	0.88 (0)	0.90 ($4\times 10^{-5}$)	0.51 ($1.1\times 10^{-2}$)	0.54 ($7\times 10^{-4}$)

**Fig. 11:**
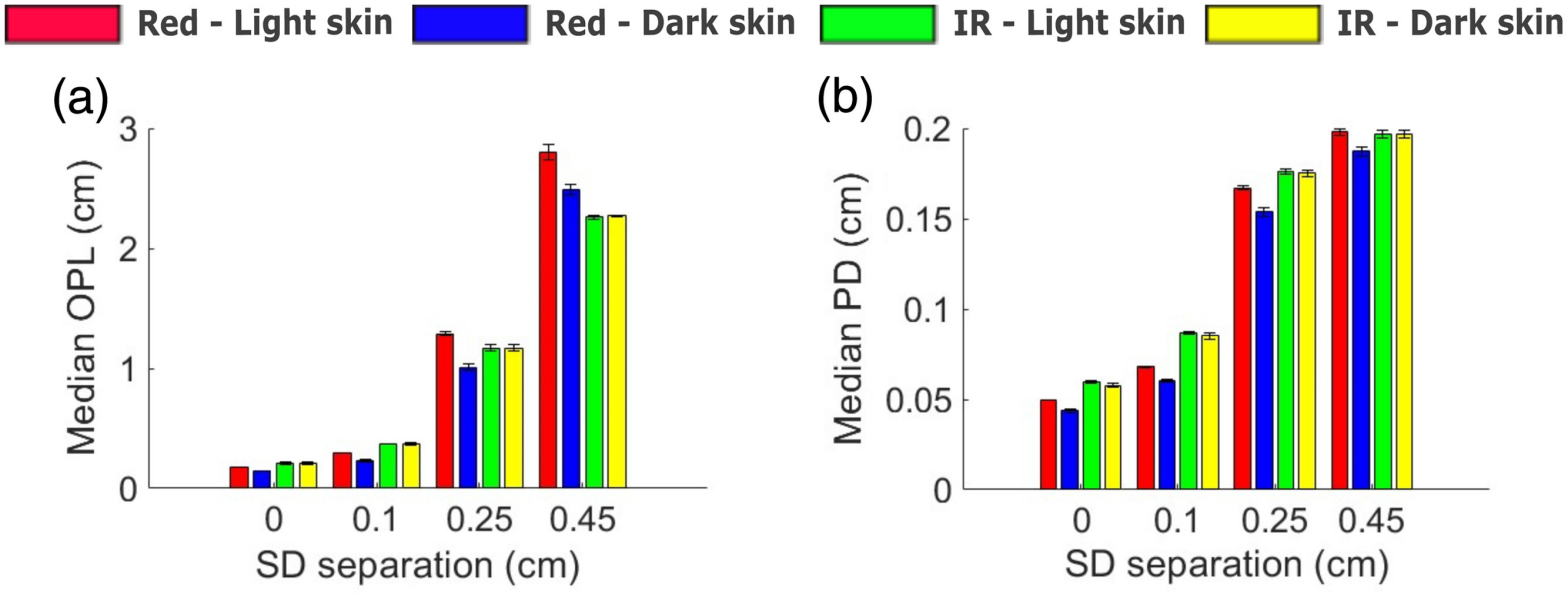
(a) Median optical path length (OPL) and (b) median penetration depth (PD) in light and dark skin against varying source–detector (SD) separations at red and IR wavelengths for ${\mathrm{SpO}}_{2}=90\%$.

Figure 11 displays the median (IQR) OPL and PD at ${\mathrm{SpO}}_{2}=90\%$ for both light and dark skin, measured at diastolic red and IR. The X-axis shows varying SD separations, and the Y-axis represents the median (IQR) of the OPL and PD in Figs. 11(a) and 11(b), respectively. Both the median OPL and median PD at red and IR increase with an increase in SD separation. At the IR wavelength, variations in median OPL between the two skin types are similar in all configurations (Euclidean distance: zero $\mathrm{SD}=1.4\times 10^{-2}\;\mathrm{cm}$, small $\mathrm{SD}=1\times 10^{-2}\;\mathrm{cm}$, medium $\mathrm{SD}=5\times 10^{-2}\;\mathrm{cm}$, and large $\mathrm{SD}=2\times 10^{-2}\;\mathrm{cm}$), whereas at red, differences in median OPL was found to be increasing with increase in SD separation (Euclidean distance: zero $\mathrm{SD}=5\times 10^{-2}\;\mathrm{cm}$, small $\mathrm{SD}=1.1\times 10^{-1}\;\mathrm{cm}$, medium $\mathrm{SD}=4.9\times 10^{-1}\;\mathrm{cm}$, and large $\mathrm{SD}=5.8\times 10^{-1}\mathrm{cm}$). At small SD separation, the median OPL in light skin at red was similar to light skin at IR (Euclidean distance: $1.2\times 10^{-1}\;\mathrm{cm}$); however, as the separation increased, the median OPL at red exceeded that of IR (Euclidean distance: medium $\mathrm{SD}=2.8\times 10^{-1}\;\mathrm{cm}$ and large $\mathrm{SD}=9.6\times 10^{-1}\;\mathrm{cm}$).

With respect to PD, IR consistently penetrated deeper than red across all SD separations as seen in Fig. 11(b). The median PD for IR was similar between light and dark skin across varying SD separations (Euclidean distance: small $\mathrm{SD}=2\times 10^{-3}\;\mathrm{cm}$, medium $\mathrm{SD}=4\times 10^{-3}\;\mathrm{cm}$, and large $\mathrm{SD}=4\times 10^{-4}\;\mathrm{cm}$). In contrast to IR, the median PD at red light in dark skin decreased due to relatively greater absorption compared with light skin (Euclidean distance: small $\mathrm{SD}=1.2\times 10^{-2}\;\mathrm{cm}$, medium $\mathrm{SD}=2.4\times 10^{-2}\;\mathrm{cm}$, and large $\mathrm{SD}=1.8\times 10^{-2}$).

## 4 Discussion

The effect of skin tones on ${\mathrm{SpO}}_{2}\text{-}R$ curves has been investigated using a four-layer finger tissue Monte Carlo simulation. The main aim of this paper was to gain insights into the origin of the problem of ${\mathrm{SpO}}_{2}$ overestimation that is frequently reported in highly pigmented subjects. Inaccurate high ${\mathrm{SpO}}_{2}$ readings can pose significant risks, resulting in delayed or denied disease detection and clinical treatment. Hence, comprehending the factors contributing to this issue is vital to enable solutions that could be devised to ensure the equitable performance of POs regardless of skin color.

Initially, a transmission mode PO configuration was studied under the effects of varying melanin in the epidermal layer. As the ${\mathrm{SpO}}_{2}$ level increased, there was a gradual decrease and increase in the detected intensities at IR and red wavelengths, respectively [Figs. 4(a) and 4(b)]. This trend was attributed to higher concentrations of ${\mathrm{HbO}}_{2}$ at elevated ${\mathrm{SpO}}_{2}$ levels, resulting in increased and decreased absorption of IR and red, respectively, thereby affecting the detected light levels. Furthermore, diastolic intensities were observed to be higher than systolic intensities at both wavelengths due to the reduced blood volume during diastole, which led to the reduction in light absorption. These trends observed in detected light intensities as a function of ${\mathrm{SpO}}_{2}$ are consistent with the findings from previous MC studies.^16^^,^^19^ As melanin’s absorption and scattering properties increased, there was a decrease in absolute light intensity at both wavelengths. However, the relative attenuation at AC red (denoted by a) and DC red (denoted by b) was greater than at AC IR (denoted by c) and DC IR (denoted by d), respectively. This indicates that melanin preferentially absorbs red light more than IR (as is observed in Fig. 3). The OPL distributions from a sample of 10,000 detected photons showed that median OPL at red decreased in dark skin, whereas in IR, it was similar to that of light skin (Fig. 5). Decrease in the median OPL at the red wavelength implies that the effect of absorption dominates the effect of scattering in dark skin. Moreover, it was observed that both the AC (difference between diastole and systole intensities) and the DC components at red and IR were relatively attenuated by the same factor (a=b and c=d) in dark skin (Table 6, transmission mode). Consequently, this uniform attenuation resulted in no change in PI for red and IR [Figs. 4(c) and 4(d)], leading to similar values for R and ${\mathrm{SpO}}_{2}$ [Fig. 4(e)].

Contrary to the results in transmission mode, in reflection mode simulation geometry, ${\mathrm{SpO}}_{2}$ was overestimated in darker skin as the optical properties in the epidermal layer varied. The detected light intensities [Figs. 7(a) and 7(b)] as a function of ${\mathrm{SpO}}_{2}$ exhibited a trend similar to that observed in transmission mode [Figs. 4(a) and 4(b)], whereas the absolute detected intensities in this geometry were significantly higher for both light and dark skin compared with those in transmission mode. This difference can be attributed to the central illumination and detection geometry, which results in shallower penetration of light within the tissue. Consequently, a greater amount of reflected light is detected. The simulations revealed that the PI at IR remained consistent between light and dark skin, whereas the PI at red exhibited variations between the two skin types [Figs. 7(c) and 7(d)]. This can be explained due to the equal/similar attenuation of AC and DC at IR (c=d) and unequal attenuation at the red in dark skin, with the AC attenuation factor exceeding the DC attenuation factor (a>b). This differential attenuation at the red wavelength (Table 6, central illumination and detection) resulted in reduced PI for dark skin compared with light skin, causing ${\mathrm{SpO}}_{2}$ overestimation [Fig. 7(e)].

To understand further the effects of SD separation in reflection mode on varying skin tones, three different SD configurations (small SD, medium SD, and large SD) were employed. The simulations illustrated that, with an increase in SD distance, the disparities in ${\mathrm{SpO}}_{2}\text{-}R$ curves due to skin color became less pronounced [Figs. 8(c), 9(c), and 10(c)]. The largest SD separation distance [Fig. 10(c)] yielded results that aligned closely with those observed in transmission mode [Fig. 4(e)]. At smaller SD distances, light penetration into the tissue is limited, causing photons to interact longer with the epidermal layer. This increased interaction resulted in relatively higher absorption and scattering of DC light at both wavelengths compared with longer SD separations. The increased scattering led to a higher amount of DC light being reflected, whereas increased absorption contributed to reduced light interaction with the arterial layer, thereby increasing AC attenuation. This effect is more pronounced at the red wavelength due to its higher optical properties of melanin, which resulted in unequal attenuation between red AC and DC (a>b). As the SD separation distance increased, so did the PD, leading to a decrease in DC light reflection (DC attenuation factor b increases) due to greater interactions along the photons’ paths to the detector, i.e., longer OPL. This phenomenon resulted in equal attenuation for both DC and AC components at red wavelengths (a=b); hence, there is no change in PI for red in dark skin relative to light skin [Figs. 10(a) and 10(b)].

It can be inferred that both the absorption and scattering effects of the epidermal layer contribute to the overestimation of ${\mathrm{SpO}}_{2}$, with absorption having a greater effect than scattering in dark skin. This is evident from the observation that median OPL and median PD decrease in dark skin relatively at the red wavelength by a greater amount across all SD separation distances compared with light skin [Figs. 11(a) and 11(b)]. If scattering dominated, then the median OPL in dark skin would be higher than in light skin at the red wavelength.

In this study, ${\mathrm{SpO}}_{2}$ overestimation in dark skin was not observed when using a transmission-based tissue model. This does not imply that melanin affects PO readings in reflection mode sensors only. Unfortunately, retrospective studies that highlighted ${\mathrm{SpO}}_{2}$ overestimation in dark-skinned individuals did not specify the PO manufacturer or whether transmission or reflection mode POs were used in their research, although it is likely that the majority would be transmission mode.^7^^,^^11^ Reflection mode sensors theoretically can be applied to any vascular area, whereas transmission mode devices are generally restricted to smaller extremities, such as toes, fingers, or earlobes.^29^ In medical settings, transmission mode POs are commonly used devices due to their high accuracy and stability compared with reflection mode.^30^ More recently, a prospective study involving healthy adult volunteers with desaturation, which used two transmission finger pulse oximeters (Masimo Radical 7 and Nellcor N-595) under low perfusion conditions, reported a missed diagnosis of hypoxemia in dark-pigmented skin types.^31^

The discrepancies between our findings and those of other published clinical studies in transmission mode geometry could be attributed to limitations in both sets of studies. In previously published clinical studies, variations in experimental protocols, differences in skin color between subjects, and the absence of information on the PO used and its type may have contributed to this difference. Alternatively, differences could be attributed to some of the following limitations.

1. The distribution of melanin in the skin is not uniform, and the amount varies within the epidermis–dermis interface. This heterogeneity affects light absorption and scattering and was not accounted for in the simulation.
2. Skin tissue contains various chromophores in addition to melanin, Hb, ${\mathrm{HbO}}_{2}$, and water, such as bilirubin, lipids, carotenoids, and different forms of hemoglobin. Exploring the complex interplay of these chromophores may provide additional insights into the effects of varying melanin.
3. Reflections due to mismatched refractive indices at air-tissue and tissue-tissue interfaces were not included.
4. A simplified four-layered tissue model may not capture all tissue layers or structures present in a real human finger. Furthermore, the geometry in this model is infinite in the x and y directions, meaning the light–tissue interactions around the edges do not accurately reflect those within a real finger. Therefore, in the future, a cylindrical model will be considered to better simulate the anatomical and geometrical features of the finger that will allow us to capture more accurate light–tissue interactions around finite-curved surfaces.
5. The error bars depicted in the simulation results do not account for other types of noise sources that typically affect physical systems, such as photodiodes. However, it was possible to compute and compare the signal-to-noise ratio (SNR) for the AC signals (${\mathrm{SpO}}_{2}=90\%$) for light and dark skin tones at red and IR wavelengths. This was carried out in a transmission mode configuration, assuming the detector is a photodiode, and the system is limited by shot noise and thermal noise. All calculations and parameters utilized for the SNR calculations^32^ are provided in the Supplementary Material. The SNR at the red wavelength was 90.5 dB in light skin and 82.4 dB in dark skin, whereas at the IR wavelength, the SNR was 102.7 dB in light skin and 98.7 dB in dark skin. Light skin exhibits a slightly higher SNR compared with dark skin at both wavelengths. Between the skin types, the IR wavelength generally provides a higher quality signal than at the red wavelength. Nevertheless, the computed SNR values at all wavelengths are well above the typical threshold of ∼10  dB, where it might be expected that a low SNR may affect the measurement. Therefore, for the cases considered in this paper, the conclusions are unaffected by noise.

## 5 Conclusion

A Monte Carlo-based finger tissue model that includes four layers has been developed to investigate the effect of varying optical properties of melanin on ${\mathrm{SpO}}_{2}$ values. The simulations were carried out in two different PO configurations: transmission mode and reflection mode. In transmission mode, uniform attenuation of AC and DC components at red and infrared wavelengths resulted in no change in the “ratio of ratios” R or ${\mathrm{SpO}}_{2}$. This does not conclude that melanin does not affect oxygen saturation readings in transmission mode. Further complex MC models are essential to confirm this result. By contrast, in reflection mode, significant variations in ${\mathrm{SpO}}_{2}\text{-}R$ curves were observed between dark and light skin tissue models. Differential attenuation of red AC and DC components and uniform attenuation of IR AC and DC components led to reduced red perfusion index and unaltered infrared perfusion index in dark skin relative to light skin. This caused R to decrease and ${\mathrm{SpO}}_{2}$ to increase. Finally, with a change in source–detector separation distance, variations in ${\mathrm{SpO}}_{2}\text{-}R$ curves due to skin tone were more significant at shorter distances. As the SD separation distance increased, the effect of skin tone on ${\mathrm{SpO}}_{2}\text{-}R$ curves diminished.

## Supplementary Material



## Data Availability

The material support for this study was obtained through the open-source Monte Carlo simulation platform MCmatlab, accessible at https://github.com/ankrh/MCmatlab. All data generated during this study are available from the University of Nottingham data repository at https://doi.org/10.17639/nott.7429.
