# Supplementary material for: Exploring the bias: how skin color influences oxygen saturation readings via Monte Carlo simulations
Source: J Biomed Opt. 2024 Aug 29;29(Suppl 3):S33308. doi: 10.1117/1.JBO.29.S3.S33308 (PMC11358849; doi:10.1117/1.JBO.29.S3.S33308)
Supplement: Supplementary file 1 [file JBO_029_S33308_SD001.docx]

Supplementary Material

**Table S1: Parameters used in SNR calculations**

| Sl No | Parameters | Values |
| --- | --- | --- |
| 1 | Input power – 940 nm | 11 mW* |
| 2 | Input power – 660 nm | 16 mW* |
| 3 | Quantum efficiency - 940 nm | 0.634* |
| 4 | Quantum efficiency - 660 nm | 0.296* |
| 5 | Bandwidth | 50 Hz |
| 6 | Feedback resistor | 100k ohms |

* Values taken from OSRAM BIOFY sensor (Version 1.2), SFH 7072 datasheet

Input values for parameters 1-4 are taken from a commonly used health monitoring chip (Osram SFH 7072). Assuming an anti-aliasing filter to remove high frequencies, the bandwidth was set to 50Hz. Typically, variable feedback resistors that range between 10k ohms and 2M ohms are employed for photoplethysmography (PPG) signal acquisition; hence, 100k ohms was chosen for this type of detector system to calculate thermal noise as an example.

**Table S2:** **Normalised intensity (%) registered on the light collector at an SpO_2_ of 90% in transmission mode configuration during diastolic and systolic cases between red and IR wavelengths, used as an attenuation factor** $\boldsymbol{(\eta}$**)**  **in the SNR calculations.**

| Skin types | Diastolic Red | Systolic Red | AC Red | Diastolic IR | Systolic IR | AC IR |
| --- | --- | --- | --- | --- | --- | --- |
| Light skin | 0.53 | 0.51 | 0.02 | 1.41 | 1.32 | 0.092 |
| Dark skin | 0.059 | 0.057 | 0.002 | 0.69 | 0.65 | 0.04 |

Normalised intensity (%) is defined as the sum of all the detected photon weights registered on the light collector normalised to the total no of incident photons (Note: the weight of an incident photon is unity; this means that the detected intensity is already normalised to the incident intensity). This term gives a direct measure of how many detected photons (in terms of weight) relative to the actual number of photons that were incident on the system.

**Example S1: SNR Calculation for Light skin at red wavelength**

**Step 1:** Calculate the incident photons/s corresponding to the defined input power (16mW)

= $\frac{P\times\lambda}{h\times c}= \frac{16\times{10}^{-3}\times660\times{10}^{-9}}{6.626\times{10}^{-34}\times3\times{10}^{8}}={5.31\times10}^{16}$photons/s

Where h is Planck’s constant and c is the speed of light in vacuum.

**Step 2:** Calculate DC detected photons/s using the attenuation factor ($\eta$) from the simulation (normalised intensity value for DC red light skin = 0.53%) and incident photons/s (calculated from step 1)

$= \eta\times incident photons/s$ = $\frac{0.53}{100}\times5.31\times{10}^{16}=2.81\times{10}^{14} photons/s$

Similarly, calculate AC detected photons/s using the attenuation factor$(\eta$) from the simulation (Diastole – Systole = 0.02% for red light skin) and incident photons/s (calculated from step 1)

$= \eta\times incident photons/s$ = $\frac{0.02}{100}\times5.31\times{10}^{16}=1.06\times{10}^{13} photons/s$

**Step 3:** Calculate DC photocurrent ($I_{DC}$), corresponding to the DC detected photons/s and quantum efficiency (Q.E.) of the detector system

$=Q.E. \times DC detected photons/s\times e$=$0.296\times2.81\times{10}^{14}\times1.6\times{10}^{-19}$

$=1.33\times{10}^{-5} A$

Where e is the charge of an electron.

Similarly, calculate AC photocurrent ($I_{AC}$), corresponding to the AC detected photons/s and Q.E. of the detector system

$=Q.E. \times AC detected photons/s\times e$=$0.296\times1.06\times{10}^{13}\times1.6\times{10}^{-19}$

$=5.02\times{10}^{-7} A$

**Step 4:** Calculate shot noise ($I_{sh}$), generated due to random nature of electron generation using $I_{DC}$, as DC photocurrent >> AC photocurrent then this dominates shot noise

$=\sqrt{2\times e\times I_{DC}\times B}$ $={1.45\times10}^{-11} A$

**Step 5:** Calculate the thermal noise ($I_{th}$), generated due to random motion of electrons in the resistor, assuming a bandwidth of 50 Hz, a feedback resistor of 100k ohms, and a temperature of 300 K.

$=\sqrt{\frac{4\times K\times T\times B}{R_{f}}}$ = 2.87×${10}^{-12} A$

**Step 6:** Calculate total noise current ($I_{total}$), which is the square root of the sum of the squares of the shot noise current ($I_{sh}$) and the thermal noise current ($I_{th}$).

$=\sqrt{\left( I_{sh} \right)^{2}+\left( I_{th} \right)^{2}}$ = ${1.48\times10}^{-11}A$

**Step 7:** Calculate SNR, assuming the lowest SNR occurs when measuring the AC photocurrent ($I_{AC}$)

$=20{log}_{10}\left( \frac{I_{AC}}{I_{total}} \right)=90.57 dB$
